# Report of the Commissioners Appointed to Inquire into the Condition of the Medical College, and Commercial Hospital and Lunatic Asylum of Ohio

**Published:** 1833

**Authors:** 


### Art. XII. Report of the Commissioners appointed to inquire into the condition of the Medical College and Commercial Hospital and Lunatic Asylum of Ohio.

In two previous numbers, we mentioned the appointment and labors of this important commission. Their report to the Legislature has just reached us. The following are the recommendations it contains.

1. That the Board of Trustees be enlarged to twenty; one of whom at least shall reside in each judicial district of the State; seven to be designated by the Board itself, as a standing committee for the transaction of ordinary business; but no professor to be appointed or dismissed, or professorship created or abolished, without a concurrence of a majority of the whole board.

2. That each judicial district (nine in number) should send two indigent pupils, as beneficiaries, to attend the Lectures in the College.

3. That the Hospital law should be so altered, as to give to the Faculty of the College, the right to prosecute *post-mortem* examinations, in their *own* way, in that infirmary.

4. That the Lunatic Asylum should be separated from the Hospital, and new buildings erected, out of the city, but so contiguous, that the professors of the Medical College might give their attendance upon its inmates.

From the late period in which this report reached us, we have only time to make a few remarks on each of these recommendations.

This is the fourth time, that the necessity of an enlarged Board of Trustees, partly resident in different portions of the State, has been urged upon the General Assembly, within the last six years. First, in the winter of 1827–8, by the Medical Convention of Ohio, meeting at Columbus; second, by the Standing Committee of the Senate on Medical Colleges and Societies, in 1830–31; third, by the Third District Medical Society, and the General Medical Society of the State, in the winter of 1832–3; fourth, by the Board of Commissioners appointed to inquire into the validity of the charges, which have at various times and by different persons, been made against the present Board. How this subject will be viewed by the present General Assembly, we do not pretend to foresee. To ourselves the case presents itself in this wise: There is much opposition to the present Trustees, and it cannot be doubted that this opposition is prejudicial to the school—a new and enlarged Board would vanquish this opposition or remove the just causes, if any exist, which have excited it.

The mode of selecting beneficiaries recommended by the Commissioners, is perhaps, since the repeal of the medical law, and the abolition of the district societies, as good as any other could be devised.

The recommendation to give to the Faculty of the College the right to prosecute *post-mortem* examinations in the Hospital, uncontrolled by its Trustees, is indispensable to its becoming a good clinical school. As the Commissioners have observed, the Trustees being elected annually, can have no settled policy on this subject; and are far less competent than the Faculty of the College, to devise a beneficial course.

What the Commissioners have said on the state of the Lunatic Asylum, is eminently entitled to the attention of the Legislature. From its foundation, it has been discreditable to the State; and until made the subject of new legislation, will continue imperfect and inadequate.

We should add, that one of the Commissioners does not concur with his colleagues in the recommendation of a new Board of Trustees for the College; and proposes, as a substitute, to make the Judges of the Supreme Court, *ex officio*, the visiters of the institution.

